# Population Genomics of Cardiometabolic Traits: Design of the University College London-London School of Hygiene and Tropical Medicine-Edinburgh-Bristol (UCLEB) Consortium

**DOI:** 10.1371/journal.pone.0071345

**Published:** 2013-08-20

**Authors:** Tina Shah, Jorgen Engmann, Caroline Dale, Sonia Shah, Jon White, Claudia Giambartolomei, Stela McLachlan, Delilah Zabaneh, Alana Cavadino, Chris Finan, Andrew Wong, Antoinette Amuzu, Ken Ong, Tom Gaunt, Michael V. Holmes, Helen Warren, Teri-Louise Davies, Fotios Drenos, Jackie Cooper, Reecha Sofat, Mark Caulfield, Shah Ebrahim, Debbie A. Lawlor, Philippa J. Talmud, Steve E. Humphries, Christine Power, Elina Hypponen, Marcus Richards, Rebecca Hardy, Diana Kuh, Nicholas Wareham, Yoav Ben-Shlomo, Ian N. Day, Peter Whincup, Richard Morris, Mark W. J. Strachan, Jacqueline Price, Meena Kumari, Mika Kivimaki, Vincent Plagnol, Frank Dudbridge, John C. Whittaker, Juan P. Casas, Aroon D. Hingorani, the UCLEB Consortium

**Affiliations:** 1 Department of Epidemiology & Public Health, UCL Institute of Epidemiology & Health Care, University College London, London, United Kingdom; 2 Department of Non-Communicable Disease Epidemiology, London School of Hygiene and Tropical Medicine, London, United Kingdom; 3 University College London Genetics Institute, Department of Genetics, Environment and Evolution, London, United Kingdom; 4 Centre for Population Health Sciences, University of Edinburgh, Edinburgh, United Kingdom; 5 MRC Centre of Epidemiology for Child Health, Department of Population Health Sciences, UCL Institute of Child Health, University College London, London, United Kingdom; 6 MRC Unit for Lifelong Health and Ageing, London, United Kingdom; 7 MRC Epidemiology Unit, Institute of Metabolic Science, Addenbrooke's Hospital, Cambridge, United Kingdom; 8 MRC Centre for Causal Analyses in Translational Epidemiology, School of Social and Community Medicine, University of Bristol, Bristol, United Kingdom; 9 Centre for Cardiovascular Genetics, Dept. of Medicine, British Heart Foundation Laboratories, Rayne Building, Royal Free and University College Medical School, London, United Kingdom; 10 Centre for Clinical Pharmacology, University College London, London, United Kingdom; 11 William Harvey Research Institute, Barts and the London. Queen Mary's School of Medicine and Dentistry, London, United Kingdom; 12 School of Social and Community Medicine, University of Bristol, Bristol, United Kingdom; 13 Division of Population Health Sciences and Education, St George's, University of London, London, United Kingdom; 14 Department of Primary Care & Population Health, University College London, Royal Free Campus, London, United Kingdom; 15 Metabolic Unit, Western General Hospital, Edinburgh, United Kingdom; 16 Genetics Division, Research and Development, GlaxoSmithKline, Harlow, United Kingdom; University College London Genetics Institute, United Kingdom

## Abstract

Substantial advances have been made in identifying common genetic variants influencing cardiometabolic traits and disease outcomes through genome wide association studies. Nevertheless, gaps in knowledge remain and new questions have arisen regarding the population relevance, mechanisms, and applications for healthcare. Using a new high-resolution custom single nucleotide polymorphism (SNP) array (Metabochip) incorporating dense coverage of genomic regions linked to cardiometabolic disease, the University College-London School-Edinburgh-Bristol (UCLEB) consortium of highly-phenotyped population-based prospective studies, aims to: (1) fine map functionally relevant SNPs; (2) precisely estimate individual absolute and population attributable risks based on individual SNPs and their combination; (3) investigate mechanisms leading to altered risk factor profiles and CVD events; and (4) use Mendelian randomisation to undertake studies of the causal role in CVD of a range of cardiovascular biomarkers to inform public health policy and help develop new preventative therapies.

## Introduction

After decades of inconsistent findings, firm genetic associations are finally being reported for risk of coronary heart disease (CHD), arterial aneurysmal disease, peripheral vascular disease and diabetes [1]–[5], and for major atherosclerotic risk factors (LDL-cholesterol [6], blood pressure (BP) [7], and smoking behaviour [8]). Moreover, variants have been identified for their associations with many other cardiovascular disease (CVD)-associated biomarkers of less certain causal relevance, including HDL-cholesterol and triglycerides [6], apolipoproteins [9], lipoprotein (a) [10], inflammation and coagulation factors [11], uric acid, and glucose [12]. This progress in cardiovascular genetics has been made possible by sequencing the human genome [13], [14], characterising the frequency and correlation between genetic variation (1000 Genomes Project and the human HapMap) and exploiting technical advances in array-based genotyping. The assembly of large case-control collections for genome wide-association studies (GWAS) using microarrays that genotype up to 1 million single nucleotide polymorphisms (SNPs), the use of stringent significance thresholds for association, and rigorous validation by replication in independent studies [15], [16], have all contributed to these major advances in genetic epidemiology.

Conservative significance thresholds required to reduce false-positive associations can lead to trait-associated variants of modest effect being overlooked in insufficiently large samples. Identifying these loci is still valuable because of the insight provided on disease aetiology and possible drug targets. To this end, multiple GWAS are being pooled by consortia usually focusing on a *single* trait or disease end-point, e.g. diabetes (DIAGRAM [5]), CHD (CARDIoGRAM [17]), stroke (the Stroke Genetics Consortium [18]), BP (the International Consortium of BP-GWAS [19]), lipids (the Global Lipids Genetic Consortium [20]), plasma glucose and related traits (MAGIC [21]), smoking behaviour (Ox-GSK [8]), and electrocardiographic QT-interval (QT-IGC). The contributing studies represent a mix of study designs, and include geographically diverse populations with differing risk factor exposures, CVD event rates, and age ranges. These studies usually provide cross-sectional disease outcome and phenotype data.

Genotyping arrays for GWAS provide broad coverage of the genome through SNPs that capture information on un-typed SNPs due to linkage disequilibrium (LD). Because of LD between variants, multiple associated SNPs are observed at each locus, mostly in non-coding DNA regions [22], which may span several genes, or may be remote from known genes. Replication usually centres on just one or two strongly associating SNPs. Consequently, determining the identity and number of causal variants is often difficult, owing to the potentially large number of unexamined variants. Fine mapping, using denser SNP association data from re-sequencing or custom gene/locus-centric SNP arrays, can help resolve causal genes and SNPs. Therefore, despite the success of GWAS, unanswered questions remain. These include: (1) the precise location of the causal SNP(s) and the gene(s) they influence; (2) the absolute (as opposed to the relative) effects of loci, and how these change with age or differing non-genetic exposures; (3) the combined influence of multiple disease- or trait-associated SNPs; (4) the full constellation of risk factors and biomarkers influenced by each SNP (pleiotropy); and (5) the precise mechanism by which many CVD-associated SNPs lead to disease.

These residual uncertainties can be addressed by higher resolution SNP typing at associated loci using gene-centric arrays, such as the Metabochip [23], in highly phenotyped, prospective cohort studies with serial risk factor/biomarker measures and information on incident disease. The Metabochip provides improved imputation accuracy, as well as greater power for detecting associations with common variants in fine mapping regions (122,241 SNPs are on the array to fine-map 257 loci which showed genome-wide significant evidence for association with one or more of the 23 cardio-metabolic traits), compared to GWAS arrays due to the increased SNP density of the Metabochip in genic regions [23], therefore offering an advantage for genetic association analyses. In addition, SNPs were selected from the databases developed by the International HapMap Project (http://www.hapmap.org) and the 1000 Genomes Project (http://www.1000genomes.org), allowing inclusion of SNPs across a wide range of the allele frequency spectrum, including those with a minor allele frequency of less than 0.1%.

The **UC**L-**L**SHTM-**E**dinburgh-**B**ristol (UCLEB) Consortium has been established to allow interrogation of genetic associations using the Metabochip. The consortium consists of 12 well-established prospective observational studies comprising over 30,000 participants: **N**orthwick **P**ark **H**eart **S**tudy **II** (NPHS II), **Wh**itehall**-II** Study (WHII), **B**ritish **R**egional **H**eart **S**tudy (BRHS), **E**nglish **L**ongitudinal **S**tudy of **A**geing (ELSA), **MRC N**ational **S**urvey of **H**ealth and **D**evelopment (MRC NSHD), **1958 B**irth **C**ohort (1958BC), **E**dinburgh **A**rtery **S**tudy (EAS), **E**dinburgh **T**ype **2 D**iabetes **S**tudy (ET2DS), **E**dinburgh **H**eart **D**isease **P**revention **S**tudy (EHDPS), **A**spirin for **A**symptomatic **A**therosclerosis **T**rial (AAAT), **Ca**erphilly **P**rospective **S**tudy (CaPS) and the **B**ritish **W**omen's **H**eart and **H**ealth **S**tudy (BWHHS). The unique properties of the consortium provide an opportunity to investigate genetic determinants of risk factors for CVD through Metabochip-wide association analyses (MWAs), as well as associations with measures of subclinical disease such as carotid intima media thickness (cIMT), and with clinical events. Integration of genotype, biomarker and disease outcome data can also provide insight into disease mechanisms and potential therapeutic targets.

## Methods and Design

### Cohorts

All 12 studies in the UCLEB consortium are UK-based with wide geographic representation. Participants are almost exclusively of European ancestry. Principal components analysis (PCA) has been used to investigate the presence of population structure and exclude outliers. The age at recruitment ranges from birth (MRC NSHD and 1958BC) to >90 years (ELSA), with most cohorts recruiting subjects in mid-life. Despite the range of ages at the time of recruitment, the current age of participants spans the 5^th^ to 9^th^ decades of life, a time at which the majority of common, non-communicable diseases manifest, rendering the consortium a valuable continuing source for cases of incident diseases. All studies have longitudinal follow up (range 5–62 years) and details of incident disease (see **Table 1**). Each of the studies has a prospective cohort design, the MRC NSHD and 1958BC also being birth cohorts. The AAAT study was designed as a RCT but the prospective follow-up allows inclusion as a cohort study. All have a DNA repository with most studies already having published genetic analyses. There are various existing collaborative links between the studies; MRC NSHD, 1958BC, ELSA, CaPS and WHII in the HALCyon network; MRC NSHD, 1958BC, BRHS, ELSA, WHII, NPHS II and EAS as the UCL Genetics Consortium, and BRHS and BWHHS as sister studies with the same sampling frame and clinic procedures.

**Table 1:** Summary of recruitment, inclusion criteria and clinical assessments in the UCLEB studies.

Study	LSHTM – Bristol based	Bristol based	UCL based							Edinburgh based				Total
	BWHHS	CaPS	MRC NSHD	ELSA	NPHS-II		1958BC	WH-II	BRHS	EAS	EHDPS*	ET2DS	AAA trial	
**Study** **design**	Prospective	Prospective	Prospective birth cohort	Prospective	Prospective		Prospective birth cohort	Prospective	Prospective	Prospective	Prospective	Prospective	Prospective	
**Sampling** **Frame**	General practices	General practices	Birth register	Respondents of HSE	General practices		Birth register	Workplace	General practices	General practices	General practices	Diabetes register (via General practices)	General practices	
**% men**	0	100	50	44	100		50	66	100	50	100	50	28	
**Baseline** **year**	1999–2001	1979–1983	1946	2002–2003	1989–1994		1958	1985–1988	1978–1980	1987–1988	1985–1988	2006–2007	1998–2001	
**N at recruitment**	4286	2512	5362	12099	3052		17416	10308	7735	1592	1592	1066	3350	**67318**
**Number of** **resurveys**	4	4	1–12 (life course) 1–4 (adulthood)	6	5 annual		9	9	8	2	2	3	Annual for CVD	
**Number of** **surveys with** **clinical data**	1	5	1	3	6		1	5	3	3	2	3	2	
**Years** **Follow up**	10	22	67	11	17		51	24	30	20	20	4	8	
**N with** **DNA**	3800^†^	1500^††^	2700^††^	5616^††^	2775^††^		8017^††^	5008^††^	3945^††^	940^††^	1200^†^	1060^†^	2833^††^	**39394**
**N with** **Metabochip**	2024	1397	2464	1982	0		5839	3408	2453	850	0	1057	0	**21474**
**N with 50k chip/custom chip**	3443	0	0	0	0		0	5456	0	0	0	0	0	**8899**
**N with GWAS**	0	0	0	8000	0		5595	0	0	0	0	0	0	**13595**
**Stored biological specimens**														
Plasma	Yes	Yes	2700	Yes		No	Yes	Yes	3945 (2)	No	No	1066	Yes	
Urine		No	2700	No		No	No	No	No	No	No	1066	No	
White cells	Yes	Yes	No	No		No	Yes	No	No	No	No	1066	No	
Lymphoid cell lines		No	2500	No		No	No	No	No	No	No	No	No	
DNA	KBio; Bristol	KBio; Bristol	GeneService; KBio	GeneService; KBio		UCL	Bristol	GeneService; KBio	GeneService; KBio	UCL; CRF Edinburgh	CRF Edinburgh	CRF Edinburgh	CRF Edinburgh	

* DNA extraction/standardisation in progress ^†^DNA extracted at baseline ^††^DNA extracted at subsequent resurvey.

Each of the studies has a defined sampling frame, inclusion criteria, and procedures for the collection and recording of demographic details, biological samples, and clinical measures. Each of the 12 cohorts has a wide range of clinical and biological measures with overlap across studies, facilitating pooled analyses with substantial power. Many have also used common or comparable measurement methods with many of the blood markers having been measured in the same laboratory. All studies follow participants for incident disease and mortality, and have an ongoing programme of clinical assessments/biological sampling.

The UCLEB consortium currently includes Metabochip information [23] augmented by imputation using the 1000 Genomes (http://www.1000genomes.org) dataset (1 million typed and imputed SNPs) from 8 studies consisting of around 21,000 samples, more than 100 phenotypes. These include a maximum of 70 blood and other biomarkers (of which 19 are available in more than 15000 participants); carotid ultrasound measures of atherosclerosis from 7200 individuals across 5 studies (MRC NSHD, WHII, BRHS, EAS and ET2DS); around 2000 cases of prevalent and incident type 2 diabetes; and almost 6000 cardiovascular events (see **Tables 2** and **3**).

**Table 2:** Measures available in the UCLEB consortium.

Number of aggregate measures	Phenotypes
>35,000	SBP, DBP, Smoking, Total Cholesterol, Fibrinogen, BMI, Height, Weight, Alcohol consumption
>30,000	LDL, HDL, Triglycerides, Social class, Physical activity
>25,000	Waist-hip ratio, HbA1c, CRP, Respiratory function (FEV1, FVC and PEFR), von Willebrand factor
>20,000	Glucose, Stress, Verbal memory, Factor VII
>15,000	D-Dimer, Educational achievement, Viscosity, ECG, Tissue plasminogen activator, IL-6, Cortisol, Short term memory, Insulin, MRC respiratory questionnaire, White cell count, Creatinine, eGFR
>10,000	Muscle function (Walking speed, Standing balance, Grip strength and Chair rises), Lp(a), Liver function (ALT, AST and GGT), ApoAI, ApoB, Mental flexibility (TMT), Homocysteine, Cognitive function (Mill Hill VS, Letter search/cancel, WMS logical/verbal memory, MMSE, Non-verbal reasoning, Processing speed (DST) and AH4)
>5,000	Digitised ECG (PR interval, QRS duration, QT interval and indices of left ventricular hypertrophy), Pulse wave velocity, Haematocrit, Prothrombin, Ferritin, IGF-1, cIMT, Cotinine, Telomere length, I-CAM, skin folds, V-CAM, ApoE, Platelets
>2,500	Arterial distensibility, Heart rate variability, ABPI, TNF-α, Bilirubin, Leptin, Dehydroepiandrosterone sulfate, Fibrin peptide A, Proteinuria, Factor VIII, Factor IX, Activated partial thromboplastin time, Activated protein C added to the Activated partial thromboplastin time, Activated protein C and Activated partial thromboplastin time, Alkaline phosphatase, Serum urea, Serum potassium, Serum sodium, Serum urate, Serum magnesium, Serum calcium, Serum phosphate, Total serum protein, Red blood cell count, Haemoglobin, Mean cell volume, Mean platelet volume, Neutrophils, Lymphocytes, Monocytes, Eosinophils, Basinophils, Vitamin C, Vitamin E, Beta-carotene, Adiponectin, IL-18, MMP-9, sCD40L, Natriuretic peptide, E-selectin, Flow-mediated dilation, Pulse-wave analysis, ApoAII

**Table 3:** Disease event definitions, incident and prevalent events by disease and medication use in the UCLEB consortium.

STUDY	Total Incident CHD	Total Prevalent CHD	Total Incident Stroke	Total Prevalent Stroke	Total Diabetes	% on Lipid Lowering drugs	% on BP drugs	% on Glucose Drugs
BWHHS	235	174	157	117	229	9%	34%	4%
CaPS	339	118	321	40	438	<1%	23%	2%
MRC NSHD	–	109	–	44	237	33%	45%	8%
ELSA	69	186	43	149	211	10%	48%	7%
1958BC*					107	1%	4%	2%
WH-II	69	206	awaiting data	85	274	12%	16%	3%
BRHS	369	422	195	109	awaiting data	10%	31%	4%
EAS	176	25	102	21	73	-	21%	3%
ET2DS	40	192	66	22	1057	85%	82%	81%
**TOTAL**	**2469**	**1323**	**1656**	**543**	**2282**			

Total incident CHD =  incident non-fatal MI or revascularization plus fatal CHD (ICD codes I20–I25, I516).

Total prevalent CHD  =  prevalent non-fatal MI or revascularization.

Total incident stroke  =  incident non-fatal stroke (ischaemic & haemorrhagic combined, but excluding TIA) plus fatal stroke (ICD codes I60×, I61×, I62, I629 I63×, I64× I65× I66×, I67, I672, I678, I679, I69×, G46×, G450, G451, G452, G453).

Total diabetes defined by a combination of self-report, medical history review, use of glucose lowering medication, or fasting glucose >7 mmol/L.

* 1958BC are currently undertaking case ascertainment.

### UCL-based studies

#### Northwick Park Heart Study II (NPHS II) [24]

From April 1989 to April 1994, 3012 healthy Caucasian men, aged 51–60 years, registered with 9 general medical practices across the UK were recruited for prospective surveillance. All eligible subjects were free of a history of unstable angina, MI or evidence of silent infarction, coronary surgery, aspirin or anticoagulant therapy, cardiovascular disease, malignancy (except skin cancer other than melanoma), or any condition precluding informed consent. Non-fasting plasma samples were taken at baseline and annually until year 6. A standard ECG was recorded and coded according to Minnesota criteria at baseline and at 6 years. Baseline plus 5 annual repeat measures are available in the entire cohort on BMI, lipids, DBP, SBP, fibrinogen and FVII. Single measures of homocysteine, Lp(a), and CRP are available. Repeat phenotypes were measured at the same centres and are complete for all those mentioned. Smoking status is known. Endpoints for CHD were fatal and non-fatal MI based on WHO criteria, silent MI or coronary revascularization procedures and sudden unexplained death. Information on fatal cancers and diabetes has also been recorded. DNA was extracted from blood samples collected in 2000.

#### British Regional Heart Study (BRHS) [25]

From 1978 to 1980, 7735 men aged 40–59 were recruited from general practices across the UK. A wide range of phenotypic measures is available for established risk markers such as lipids, blood pressure and inflammatory and haemostatic markers. Most of these measures were taken both at recruitment and re-examination, which occurred in 1998–2000 when men were aged 60–79. At this re-examination 4252 participants attended and DNA was extracted for 3945. A case-control sample was selected using 1095 cases with prevalent data at re-examination or incident cases of CHD or stroke over the next 8 years and 1358 controls. The controls were frequency matched based on being in the same town and within the same fixed 5-year age band as the cases. Data on important behavioural variables such as cigarette and alcohol consumption, as well as physical activity, have been regularly collected through follow up. Well validated outcome variables such as major coronary heart disease, stroke, diabetes, and revascularisation, as well as cause-specific mortality, continue to be collected from medical records almost 30 years after recruitment.

(http://www.ucl.ac.uk/pcph/research-groups-themes/brhs-pub).

#### Whitehall II Study (WHII) [26]

Whitehall II recruitment of 10,308 participants (70% men) between 1985 and 1988 involved 20 London based Civil service departments. Genetic samples were collected in 2004 from over 6,000 participants. The study is highly phenotyped for cardiovascular and other ageing related health outcomes, with 9 phases of follow up (5 with clinical assessment and biological sampling), over 20 years of follow up. A wide variety of health behaviour and environmental data are also collected and the participants are consented for linkage to recorded clinical data such as Hospital Episode Statistics (HES), the Office of National Statistics mortality data and the national registry of acute coronary syndromes in England and Wales (Myocardial Ischaemia National Audit Project).

(http://www.ucl.ac.uk/whitehallII/).

#### English Longitudinal Study of Ageing (ELSA) [27]

This is a national cohort of participants (48% men) aged over 50 years recruited from the Health Surveys for England in 1998, 1999 and 2001. Genetic data were collected at Wave 2 of the study (2004/5). A wide range of phenotypic measures relevant to ageing are available. These measures were made at Wave 0 of the study (1998, 1999 and 2001) and at follow up (2004/5). Data on health behaviours and a wide range of health outcomes are available. Nearly all participants (97%) are also consented to linkage to routine data such as HES, which allows for the assessment of health outcomes and cause specific mortality. A case-control sample was selected using 412 cases and 1573 controls. Controls and cases were matched by sex and 5-year age bands at Wave 2.

(http://www.ifs.org.uk/elsa/).

#### Medical Research Council National Survey of Health and Development (MRC NSHD) [28]

This is an on-going prospective birth cohort study consisting of all births in England, Scotland and Wales in one week in March 1946. The sample includes single, legitimate births whose fathers were in non-manual or agricultural occupations and a randomly selected one in four of all others, whose fathers were in manual labour. The original cohort, now 66 years of age, comprised 2,547 women and 2,815 men who have been followed-up over 20 times since their birth. The data collected to date include repeat cognitive function, physical, lifestyle and anthropomorphic measures, as well as blood analytes and other measures. The cohort recently completed a particularly intensive phase of clinical assessment and biological sampling with blood and urine sampling and analysis, and cardiac and vascular imaging [29]. DNA was extracted from blood samples collected in 1999. Follow-up for disease outcomes is by self-reports of doctor diagnosed events that have been validated against General Practice (GP) records.

(http://www.nshd.mrc.ac.uk/).

#### 1958 Birth cohort (1958BC) [30]

1958BC is a prospective birth cohort study consisting of all births in England, Scotland and Wales in one week in March 1958. Participants to the cohort have been followed-up regularly since birth with prospective information collected on a wide range of indicators related to health, health behaviour, lifestyle, growth and development. There have been 9 contacts with the participants since their birth (ages 7, 11, 16, 23, 33, 41, 45, 47, and 50 years). The biomedical survey at age 45 years included collection of blood samples and DNA from about 8000 participants. Immortalised cell lines providing an unlimited resource for future genetic studies have been created for 7500 participants. Follow-up for disease outcomes is by self-reports of doctor diagnosed events and record linkage for fatal events.

### Bristol/London School-based studies

#### Caerphilly prospective study (CaPS) [31]

This study is based on men aged between 45 to 59 years who resided in the small South Wales town of Caerphilly between the examination dates of 1979 &1983. Of the 2818 eligible, 2512, (89%) were recruited. The men were studied at baseline (Phase 1) and each subsequent 5 year period (Phase 2–5) and have therefore been followed up for around 20 years. An additional 447 patients were recruited at phase 2. The cohort has a wide range of cardiovascular phenotypes and at phase 3, cognitive function was also assessed, which has been supplemented with clinical dementia and cognitive impairment at phase 5. DNA was extracted from blood samples collected in 1992–1994. Follow-up for disease outcomes is by self-report from participants, who are also linked to hospital episode discharge summaries for validation checks to comply with WHO criteria, as well as death certificates for fatal events.

(http://www.bris.ac.uk/social-community-medicine/projects/caerphilly/about/).

#### British Women's Heart and Health Study (BWHHS) [32]

Established in 1999 as a study of women to parallel the BRHS, it used the same sampling frame and very similar clinic protocols to the 20 year follow-up of BRHS. From 1999–2001, 4286 women aged 60–79 were randomly selected from 23 general practices across the UK. A wide range of phenotypic measurements were obtained at baseline including anthropometry, blood pressure, ECG, lung function tests and fasting blood samples. Glucose, insulin, lipids, clotting and inflammatory markers have been assayed and stored serum is available. DNA was extracted from blood samples collected at baseline in 1999–2001. Data on sociodemographic and lifestyle variables such as cigarette smoking, alcohol consumption, diet, physical activity, reproductive health, education, occupation, quality of life, and activities of daily living have been collected repeatedly (baseline, years 3, 7, and 12). Follow-up for disease outcomes is by biennial medical record review (with validation checks) and cancer registrations and death certificates obtained from the National Health Service (NHS) Central Registration. Detailed follow-up is collected on coronary heart disease, stroke, type 2 diabetes, pulmonary embolism, deep vein thrombosis and cancer events. The UCLEB case-control sample was selected using 523 cases with prevalent disease at recruitment or incident cases of CHD or stroke up to 2010. 1501 controls were frequency matched based on being in the same town and within the same fixed 5-year age band as the cases.

(http://www.lshtm.ac.uk/eph/ncde/research/bwhhs/index.html).

### Edinburgh-based studies

#### Edinburgh Artery Study (EAS) [33]

At baseline (August 1987-September 1988), an age-stratified random sample of men and women, aged 55–74 years, was selected from the age-sex registers of ten general practices with catchment populations spread geographically and socioeconomically throughout the city of Edinburgh. Subjects were excluded if they were unfit to participate (e.g. due to severe mental illness or terminal disease). These exclusions were replaced by other randomly sampled subjects. The study population is almost exclusively European. DNA was extracted at 5 years follow-up. Physical examinations were performed by specially trained research nurses using standardised operating procedures. The quality of clinical measurements were checked before and during the study by repeat measurements taken intermittently by the study co-ordinator. Individual observer measurements were assessed for drift. Blood assays were performed in accredited laboratories using international standards. Subjects have been followed up for 20 years for cardiovascular events, using repeat self-reporting questionnaires, record linkage for hospitalisations and deaths, and validation of events against pre-specified criteria through searching of hospital and GP notes. Comprehensive clinical examination was repeated at 5 and 12 years after commencement of the study, resulting in repeat measurements of several key variables.

#### Edinburgh Heart Disease Prevention Study (EHDPS) [34]

At baseline (1985–1987), all men aged 30–59 years and registered with one of two general practices in the city of Edinburgh, were invited to participate in this study. The response rate was 69% and follow-up of non-responders showed that there was no substantial bias. DNA was extracted at baseline. Physical examinations were performed by a specially trained research nurse using standardised operating procedures. Blood assays were performed in accredited laboratories using international standards. Subjects have been followed up after 20 years for cardiovascular events, using repeat self-reporting questionnaires and record linkage for hospitalisations and deaths.

#### Edinburgh Type 2 Diabetes Study (ET2DS) [35]

At baseline (August 2006 to August 2007), an age-stratified random sample of men and women with type 2 diabetes, aged 60–74 years, was selected from the Lothian Diabetes Register (LDR), a comprehensive database of subjects with known type 2 diabetes living in Lothian. Subjects were excluded if they did not meet WHO criteria for type 2 diabetes, or if they were physically unable to complete the clinical and cognitive examination. The study population is almost exclusively European. DNA was extracted at baseline. Physical examinations were performed by specially trained research nurses using standardised operating procedures. The quality of measurements was checked using observation of research staff by study investigators and inter-observer variability assessments were made for key variables. Blood assays were performed in accredited laboratories using international standards. Retrospective data on cardiovascular disease and selected physical and biochemical variables were retrieved using record linkage for hospitalisations and deaths since 1985 and using data from the LDR. Subjects returned for further clinical examination after one year and were examined again after they had participated for 4 years.

#### Asymptomatic Atherosclerosis Aspirin Trial (AAAT) [36]

At baseline (1998 to 2000), all men and women aged 50–75 and registered with participating general practices spread throughout Edinburgh, Glasgow and Lanarkshire (83% of all practices within study area), were invited to participate in this study. Subjects were excluded if they had a history of MI, stroke, angina or peripheral arterial disease (PAD) and included if they had an ankle brachial index of 0.9 or less. They were therefore a healthy, but moderately increased risk population, in terms of cardiovascular disease. Subjects were further excluded if they were currently using aspirin or other antiplatelet or had a contraindication to aspirin therapy. Subjects were followed up for 8 years for cardiovascular events, using annual contacts with subjects, record linkage for hospitalisations and rigorous validation of events against pre-defined criteria using hospital and GP notes. Physical examination was performed at baseline and after 5 years by specially trained research nurses using standard operating procedures, and blood assays were performed in accredited laboratories using international standards. DNA was extracted 3 months after recruitment.

### Work flow and organisation

The workflow developed to co-ordinate genotyping, merge and collate with harmonised phenotype and disease end-point data is summarised in **Figure S1**.

### Organisation and governance

Informed consent was obtained for all subjects included in UCLEB research. Written approval from individual Research Ethics Committees to use anonymised individual level data has been obtained by each participating study. All data obtained and generated within the UCLEB consortium are fully anonymised, contain no personal identifiers and adhere to the contributing studies restrictions on deductive disclosure. Senior investigators from each participating study were invited to join the UCLEB steering group to discuss key projects to be carried forward and to assess the potential for external collaborations. An analysis group meets weekly focusing on methods development and analyses. There are regular weekly teleconferences organised from the coordinating centre at UCL during which the whole consortium is updated on various aspects of genotype and phenotype data management, and on-going and proposed analyses. An access-restricted Google-hosted website has been set up to facilitate sharing of minutes, analysis plans, project proposals and other useful resources. The cohorts included in the UCLEB consortium have individual policies and mechanisms for data sharing and all have an excellent track record in this regard. Most of the studies have contributed to the highly cited Emerging Risk Factors Collaboration reports on CV risk factors and biomarkers, and to GWAS consortia and other large-scale genetic discovery efforts. Opportunities for data sharing are maximised because requests to individual studies are also possible, e.g. for projects that focus on certain study designs or where outcomes are available only in a subset. The proposals to access UCLEB data are evaluated by members of the steering committee using a standardised data request form. Aggregate SNP data for specifically requested traits are shared with external collaborators according to the external project analysis plan.

### Genotyping

Around 21,000 individuals across the cohorts have been typed using Metabochip (see **Table 4**), a genotyping platform consisting of ∼200,000 SNPs, which cover the loci identified by GWAS in cardiometabolic diseases, and rare variants from the 1000 Genomes Project [23]. This will be supplemented by SNP data from a whole genome array in the 1958 Birth Cohort, the 50 k HumanCVD Beadchip [37] in WHII and BWHHS, and prior candidate and GWAS replication work in all studies. The NPHS-II, EHDPS and AAAT studies are available for new bespoke genotyping e.g. to validate associations from UCLEB samples genotyped by Metabochip. This will yield a powerful aggregate dataset rich in genetic and phenotypic detail.

**Table 4:** Information on genotyped and imputed SNPs.

	WHII (UCL Genomics)*	WHII (Cambridge)*	CaPS	EAS	ET2DS	BRHS	BWHHS	MRC NSHD	1958BC	ELSA
**Genotyping**										
Array Reader	iScan	BeadArray	iScan	Beadstation S225	iScan	iScan	iScan	BeadArray	iScan	iScan
GenomeStudio Version	v2010.1	v2010.1	v2010.3	v2009.1	v2010.3	v2010.3	v2010.3	v2010.1	NA	v2010.3
Clustering Algorithm	GenTrain, followed by reclustering based on data	Custom – using top 100 samples to create cluster file and apply it to the set.	GenTrain, followed by reclustering based on data	GenTrain 2.0, followed by reclustering based on data	GenTrain, followed by reclustering based on data	GenTrain, followed by reclustering based on data	GenTrain, followed by reclustering based on data	Custom – using top 100 samples to create cluster file and apply it to the set.	GenTrain, followed by reclustering based on data	GenTrain, followed by reclustering based on data
GenCall Threshold	0.15	0.15	0.15	0.15	0.15	0.15	0.15	0.15	0.15	0.15
Allele Format	Plus Strand	Plus Strand	Plus Strand	Plus Strand	Plus Strand	Plus Strand	Plus Strand	Plus Strand	Plus Strand	Plus Strand
Total SNPs	196725	196725	196725	196725	196725	196725	196725	196725	196725	196725
Total Samples	1008	2405	1411	863	1075	2454	2068	2488	5840	2007
Sample Call Rate threshold	0.95	0.95	0.95	no QC on supplied data	0.95	0.95	0.95	0.95	0.95	0.95
Samples passing call rate 0.95	1005	2388	1376	814	1050	2381	1994	2475	5813	2004
Samples passing all QC	3078		1349	764	1007	2342	1980	2464	5560	1883
SNP Call Rate threshold	0.95		0.95	no QC on supplied data	0.95	0.95	0.95	0.95	0.95	0.95
SNPs passing call rate 0.95	193203		192040	186324	192701	185419	190386	150443	183675	191478
**Post-Imputation**										
No. polymorphic SNPs with Rsq ≥0.3	3838657		4097468	3967944	4099771	4009987	4079402	4113227	3864584	4085542
No. polymorphic SNPs with Rsq ≥0.8	1217802		1309437	1265020	1312849	1271204	1298293	1311791	1302863	1303682

* The WHII study was typed at two centres with different array readers, which were compared and shown to be concordant.

Duplicate samples have been genotyped to compute the error rate. Initial quality control analysis on genotyped data identified any problem samples that have been subsequently excluded in further analysis. These included: checks for discordance between reported and genetically-determined ethnicity, replicate concordance, sample mix-up (unknown duplicates and comparison to previously genotyped data where available), gender ambiguity and cryptic relatedness (see **Figure S1**).

### Imputation using data from 1000 Genomes

Although coverage of the genome is less comprehensive using the Metabochip than a whole genome array, imputation against the 1000 genomes European ancestry reference sample extends coverage from 200,000 to approximately 1 million SNPs, when the R^2^ is ≥0.8 (see **Table 4**), with dense coverage of loci of interest for cardiometabolic disease, including approximately 70% of the druggable genome. Imputation using Metabochip data served to fine-map in and around regions covered by the array. However, the gene-centric nature of Metabochip means that there are extensive intergenic regions of the genome that have no SNP coverage, and therefore, imputation provided little additional information in these regions. Our imputation process was based on the strategy summarised at: http://genome.sph.umich.edu/wiki/Minimac:_1000_Genomes_Imputation_Cookbook, and proceeded in three distinct stages: chunking, phasing and imputing.

### Chunking

To speed up the overall imputation process, the genome was broken into overlapping chunks specified by reference to the physical map. Each chunk consisted of 1000 consecutive SNPs and consecutive chunks overlapped by 250 SNPs. The final chunk in each chromosome consisted of any remaining SNPs (less than 1000) plus a 250 SNP overlap with the penultimate chunk.

### Phasing

Each chunk was phased using the program MACH1 (downloaded from: http://www.sph.umich.edu/csg/abecasis/MACH/download/), which implements a Markov Chain Monte Carlo (MCMC) Haplotyping algorithm [38], [39]. The phasing process can be set to consider all possible haplotypic states for the genotypes but this is too computationally intensive to be practical. We therefore phased each chunk considering 500 states and the number of rounds of MCMC was set to 30.

### Imputation

Phased haplotypes were used as a basis for imputation of untyped SNPs using the method described by Li *et al*. 2010 [39], which used an external set of reference haplotypes (1000 genomes, February 2012 release, CEU haplotype set) to infer the most likely allele call for untyped loci. The method was implemented using the software Minimac (downloaded from: http://www.sph.umich.edu/csg/cfuchsb/minimac-beta-2013.7.17.tgz. Following imputation, chunks were reassembled into full chromosomes. The genotypes for SNPs that lay in overlapped regions were taken from the chunk in which the R^2^ statistic for imputation quality was greatest.

The UCLEB data was collated in a dosage format, which is readily utilised by the analytical package PLINK [40], as well as in probability format that can be used by the R Package snpStats (downloaded from: http://www.bioconductor.org/packages/2.10/bioc/html/snpStats.html), which offers a useful range of analytical functions coupled with the flexibility of the R programming environment for data manipulation and further analysis.

### Phenotype and disease endpoints

Phenotypic data available for sharing from each study were harmonized to agreed units and categories determined by individual study and phenotype data managers. Data were then collated at the UCLEB coordinating centre (**Table 3**). Disease definitions were as follows: CHD is all non-fatal myocardial infarction (MI) or any revascularisation procedure (coronary artery bypass surgery or angioplasty) and fatal CHD. Stroke is all non-fatal stroke (ischaemic & haemorrhagic combined) excluding TIA and fatal stroke. Fatal events are defined or matched according to a pre-defined list of ICD codes. Type II diabetes is based on either self-reported, medical history review, taking glucose lowering medication, or a fasting glucose >7 mmol/L. Medication data were also collated, including those on lipid lowering drugs (statins or other medication), blood pressure lowering drugs, and glucose drugs (**Table 3**).

## Analyses

Analysis plans have been written that can be adapted by each working group for specific analyses, and Stata, PLINK and R syntax are available for these analyses. In order to address the core aims of UCLEB, five broad analytical approaches will be applied.

- Metabochip-wide genetic discovery analysis to be run individually on each study, from which study-specific estimates will be pooled by fixed effects meta-analysis weighting by inverse variance using the METAL program, as well as by random effects meta-analysis, to generate summary estimates for each trait. Medication use for the main drug classes affecting cardiometabolic traits (cholesterol, blood pressure and glucose) has been carefully recorded. Previously validated methods [41], [42] for adjusting genetic associations for medication use will be applied. Heterogeneity will be estimated by Cochran's Q statistic and the I^2^ value (and 95% confidence interval; CI). SNPs most likely to mark or represent causal sites will be identified using variable selection methods, as well as conditional analyses to identify independent SNPs at each locus applying locus-specific Bonferroni correction.
- Analysis of multiple phenotypes (phenome scans) for improving power and investigating pleiotropy. Considering the extra information provided by the covariance between the traits allows the joint analysis of multiple phenotypes, in order to increase power. One such implementation is MultiPhen [43], in which the genotype of each SNP is regressed on a group of phenotypes. Pleiotropy will be explored in two ways; (i) using Bayesian statistical methodology, where the association between multiple phenotypes and multiple SNPs will be analysed simultaneously using the GUESS software [44]; and (ii) through phenome scans based on associations reported in prior studies, where the association of a selection of known disease associated genetic loci is tested for the large number of available phenotypes in the UCLEB data to establish common pathways and functional links.
- Application of genetic associations in risk prediction models. The absolute, relative and attributable genetic risk of CVD events (overall, and separately for fatal/non-fatal CHD and stroke) will be estimated and compared with the associations of non-genetic risk factors using SNPs identified by prior GWAS that are included in Metabochip. Analyses will use Cox proportional hazards models and derived hazard ratios (HRs). Risks will be estimated within study and pooled by random effects meta-analysis with exploration of heterogeneity and effect modification by strata of age, CVD risk factors, and by geographic region. We will generate standard prediction metrics (e.g. C-statistics, net-reclassification, integrated discrimination index) that will compare the benefit of genotypes for CVD prediction with established non-genetic risk scores. The large number of incident CV events in the UCLEB consortium (>5000) will allow precise risk estimates to be obtained and minimise potential false-positive findings.
- Mendelian randomisation (MR) analyses. MR studies typically quantify and compare three associations: (i) biomarker-disease in prospective cohort studies; (ii) genotype-biomarker in cross-sectional or prospective cohort studies; and (iii) genotype-disease in prevalent case-control studies or studies of incident cases and controls nested within a cohort study. Triangulation of the risk estimates provides evidence on causation, with the magnitude of the causal association being estimated by instrumental variables regression. Despite emerging successes of MR, two limitations remain. First, many biomarker-associated SNPs are of weak effect, compromising power and necessitating large sample sizes. Second, although SNPs identified for an index association with a single CVD risk factor/biomarker are not generally associated with exogenous exposures influencing CVD risk (e.g. diet, physical activity, socioeconomic status), they are frequently associated with a diverse range of endogenous biomarkers. This can compromise interpretation of an MR analysis based on a single locus. The issue of power is addressed in UCLEB both through use of gene scores and through external collaboration with other studies and consortia to ensure case numbers are not limiting. The use of genetic instruments comprising SNPs from different genetic loci, each independently associated with the biomarker of interest, increases power, since each SNP contributes incrementally to the marker variance, and also helps reduce non-specificity, because the relative genetic effect on traits other than biomarker of interest tends to attenuate.
- Genetic effects on risk factor/biomarker trajectories. Analysis of risk factor trajectories will involve generation of standard deviation scores for each trait, cross-sectional descriptive analyses at different ages, and the development of hierarchical mixed models which account for correlation between repeated measures, as well as age-by-genotype interaction tests. We will use multiple imputation for the management of missing data.

**Figure S2** gives additional information on the analyses that have been prioritised by UCLEB.

### Power calculations

Power and sample size calculations were conducted to evaluate the ability of the UCLEB datasets to discover novel loci and to test the effect of loci validated for an index trait on other traits and outcomes. Conservative alpha values of 1×10^−7^ (GWAS level) were adopted for the former and 10^−4^ for the latter. All sample size estimates correspond to the total number of individuals and assume a 10-year CVD event rate of 16%, based on average age, follow-up period and gender of participants in UCLEB cohorts.

#### Quantitative traits

For quantitative traits, a good approximation for the sample size is given by N = (z_α_+z_β_)^2^/q^2^ where z_α_ and z_β_ are values of the standard normal distribution for specified alpha and beta error and q^2^ is the amount of variance explained, which is determined by effect size and minor allele frequency in the case of single SNPs. In addition to alpha and sample size, power depends on the variance explained by a SNP, which is in turn related to minor allele frequency (MAF, p_Au_) and effect size (d) and is given by q^2^ = 2p_Au_(1-p_Au_)d^2^. **Tables S1a** and **S1b** present the resulting power estimates in order to be able to discover SNPs that explain as little as 0.5% of the variance with 80% power. The tables provide power for sample sizes in the range 2,500 and 20,000 to detect variance in a quantitative trait of 0.5–5% at a p-value of 10^−7^ (**Table S1a**), and a p-value of 10^−4^ (**Table S1b**).

#### Events

For disease events, power depends additionally on incidence rate. A differentiation is made between common alleles (see **Tables S2a** and **S2b**) and rare alleles (see **Tables S2c** and **S2d**). Higher MAFs are often accompanied by smaller effect sizes while rare alleles have a higher likelihood of larger effects. As illustrated by **Tables S2a–d**, the combination of very small MAF and very small effect leads to a substantial inflation of the effect of sample size on power. With an overall sample of 30,000 subjects, and a 16% 10-year event rate, power estimates mostly exceed 80% for a range of plausible effect sizes. For example, with a total sample of 27,323 individuals among whom the 10-year event rate is 16%, power to detect a common SNP with MAF of 15% and odds ratio of 1.2 is 80%. With regard to rare alleles that have a frequency of only 1%, achieving a power of 80% for an odds ratio of 2, requires 18,456 subjects.

## Discussion

UCLEB is a large-scale epidemiological resource bringing together diverse expertise across studies, scientific advisors, phenotype experts, statistical experts and analysts. UCLEB has an established research governance structure, data sharing arrangements, steering, operations and analysis groups, a centralised, secure, access-managed genetic and phenotypic data repository, and agreed analysis protocols. The strength of cohort-based analyses is that genetic loci can be identified for every quantitative trait recorded in sufficiently large numbers. UCLEB has more than 100 traits indexing 16 organs and biological systems. Moreover, as well as the established 1-trait: 1-Metabochip-wide analysis model, there is an opportunity to integrate Metabochip information, for example, to identify quantitative traits with overlapping genetic regulation and genetic loci with effects on multiple pathways.

Heritability estimates provide an indication of the overall genetic contribution, but not the number of genes influencing a trait or the size of effect. Despite similar heritability, genes for some traits such as blood inflammation markers [45], [46] have been identified by moderately-sized GWAS, while others such as BMI [47] and height [48] have required much larger data sets to identify genes of smaller effect with statistical confidence. In general, traits more proximal in the pathway from genome variation to disease (e.g. blood proteins and metabolites) are likely to be identified with smaller sample sizes than distal (higher order) phenotypes such as ECG parameters and BP. However, expansion of the population resources in both domains would be beneficial even for traits where some genetic loci have already been identified (e.g. LDL-cholesterol), because of the large residual unexplained phenotypic variance. For the majority of traits in this consortium, the genetic determinants have yet to be fully characterised.

By exploiting measures of continuous phenotypes where available, case-control GWAS have not only provided insight on disease-associated loci but have also started to uncover genetic effects on quantitative traits. However, highly-phenotyped, population-based prospective studies are particularly suited for genetic analysis of quantitative traits because of: (1) the range of measures; (2) longitudinal follow-up that also allows exploration of genetic effects on trajectories, and at critical periods; (3) the addition of new phenotypic detail with new waves of resurvey and data collection, which enrich the resource, so that the value of the investment in genotyping extends beyond any single trait or outcome; (4) assessment of context dependent genetic effects because of exhaustive information on diet, records (and sometimes objective measures) of smoking and physical activity, as well as education, employment, medication and many other environmental factors [49], [50]; and (5) repeated measures not only of many of the outcomes of interest but also some of the important environmental modifiers (e.g. smoking, alcohol consumption, physical activity and social indices), which will help improve precision by allowing control for regression dilution bias, and will facilitate analysis of gene-environment interaction [51]. Together with the use of the Metabochip SNP array that more densely captures genome variation, including rare and copy number variants, this should allow more comprehensive evaluation of genetic effects on disease-relevant traits.

A number of large consortia have already been assembled to discover and subsequently fine map genetic associations of cardiometabolic traits and disease end points [52]–[54] using Metabochip. UCLEB studies have already contributed to some of these efforts, but these are typically based on sharing of summary level data on a narrow range of phenotypes or disease end-points. The added value of the UCLEB consortium comes from the ability to undertake discovery of the genetic loci for less commonly available phenotypes not currently the subject of consortium based efforts, extend the associations of known cardiometabolic loci to a wider range of phenotypes (phenome scans), to investigate the effects of known genetic variants on biomarker trajectories and variability (where repeat measures are available), to evaluate the predictive utility of known disease associated genetic variants for incident CVD events, and to generate and optimise genetic tools for Mendelian randomisation analyses based on effect size and specificity for the trait of interest. In this respect, the UCLEB is most similar in design, organisation, exposure measures and aims to two other assemblies of population based studies engaged in collaborative genomic research. These are the National Heart Lung and Blood Institute Candidate Gene Association Resource (CARe) [55], and the Cohorts for Heart and Aging Research in Genomic Epidemiology (CHARGE) Consortium [56].

## Conclusions

In conclusion, UCLEB provides a stable, long-term resource for large-scale, integrated genomics analyses, with the potential to add proteomic and metabolomic technologies as they emerge. The archived biological samples will facilitate more comprehensive phenomic analysis to match the breadth of genomic data. The integration of multiple layers of –omics data within the framework of cohort studies should eventually lead to a more comprehensive understanding of the mechanisms of common disease [57]. We recognise that very large sample sizes are required for assessments of gene-environment interactions (more so for disease outcomes than quantitative traits) and are likely to require “consortia of consortia”. Therefore, UCLEB will also continue to collaborate externally and contribute to the wider research efforts focused around specific diseases and phenotypes. UCLEB will do this by building on an already extensive network of successful links with other investigators in the UK and internationally, that will permit powerful large-scale analyses.

## Supporting Information

Figure S1 **UCLEB workflow.**
(DOCX)

Figure S2 **Prioritised analyses in UCLEB.**
(DOCX)

Table S1 Table S1a: Power for discovery for quantitative traits. Table S1b: Power for translation for quantitative traits
(DOCX)

Table S2 Table S2a: Power for discovery for common alleles. Table S2b: Power for translation for common alleles. Table S2c: Power for discovery for rare alleles. Table S2d: Power for translation for rare alleles.
(DOCX)
