# Correction: Population Genomics of Cardiometabolic Traits: Design of the University College London-London School of Hygiene and Tropical Medicine-Edinburgh-Bristol (UCLEB) Consortium

**DOI:** 10.1371/annotation/89b51e89-a415-49c7-9caa-8dfcf6fde855

**Published:** 2013-09-24

**Authors:** Tina Shah, Jorgen Engmann, Caroline Dale, Sonia Shah, Jon White, Claudia Giambartolomei, Stela McLachlan, Delilah Zabaneh, Alana Cavadino, Chris Finan, Andrew Wong, Antoinette Amuzu, Ken Ong, Tom Gaunt, Michael V. Holmes, Helen Warren, Teri-Louise Davies, Fotios Drenos, Jackie Cooper, Reecha Sofat, Mark Caulfield, Shah Ebrahim, Debbie A. Lawlor, Philippa J. Talmud, Steve E. Humphries, Christine Power, Elina Hypponen, Marcus Richards, Rebecca Hardy, Diana Kuh, Nicholas Wareham, Yoav Ben-Shlomo, Ian N. Day, Peter Whincup, Richard Morris, Mark W. J. Strachan, Jacqueline Price, Meena Kumari, Mika Kivimaki, Vincent Plagnol, Frank Dudbridge, John C. Whittaker, Juan P. Casas, Aroon D. Hingorani, the UCLEB Consortium

Author Daniel I Swerdlow was not listed in the author byline. This author should be the seventeenth author, and be affiliated with Department of Epidemiology & Public Health, UCL Institute of Epidemiology & Health Care, University College London, London, United Kingdom. The contributions of this author are: Analyzed the data.

Author Claudia Langenberg was not listed in the author byline. This author should be the thirty-third author and affiliated with Department of Epidemiology & Public Health, UCL Institute of Epidemiology & Health Care, University College London, London, United Kingdom. The contributions of this author are: Performed the experiments, contributed reagents/materials/analysis tools.

The correct version of the Funding statement is: "The UCLEB Consortium is supported by a British Heart Foundation Programme Grant (RG/10/12/28456). NPHS-II is supported by the UK Medical Research Council, the United States National Institutes of Health (grant NHLBI 33014) and Du Pont Pharma, Wilmington, USA. BRHS is a British Heart Foundation Research Group and is supported by British Heart Foundation (RG/04/003). The WHII study is supported by grants from the Medical Research Council (G0902037; ID85374), British Heart Foundation (RG/07/008/23674), Stroke Association, National Heart Lung and Blood Institute (5RO1 HL036310), National Institute on Aging (5RO1AG13196) Agency for Health Care Policy Research (HS06516), and the John D. and Catherine T. MacArthur Foundation Research Networks on Successful Midlife Development and Socio-economic Status and Health. Samples from the ELSA DNA Repository (EDNAR), received support under a grant (AG1764406S1) awarded by the National Institute on Ageing (NIA). ELSA was developed by a team of researchers based at the National Centre for Social Research, University College London and the Institute of Fiscal Studies. The data were collected by the National Centre for Social Research. MRC NSHD is funded by the UK Medical Research Council. DNA collection of the 1958BC was funded by the UK Medical Research Council (G0000934) and the Wellcome Trust (Grant 068545/Z/02). Genotyping was supported by a contract from the European Commission Framework Programme 6 (018996) and grants from the French Ministry of Research. BWHHS is supported by funding from the British Heart Foundation and the Department of Health Policy Research Programme (England). EAS is funded by the British Heart Foundation (Programme Grant RG/98002), with Metabochip genotyping funded by a project grant from the Chief Scientist Office of Scotland (Project Grant CZB/4/672). AAAT was funded by the British Heart Foundation (Programme Grant RG/97006), the Wellcome Trust (Project Grant 057762), the Chief Scientist Office of Scotland (Project Grant K/OPR/2/2/D320), Chest Heart and Stroke Scotland (Project Grant Res03/A75) and Bayer plc (Unrestricted Investigator Led Grant). Research clinics were held at the Wellcome Trust Clinical Research Facility in Edinburgh. ET2DS is funded by the Medical Research Council (Project Grant G0500877), the Chief Scientist Office of Scottish (Programme Support Grant CZQ/1/38), Pfizer plc (Unrestricted Investigator Led Grant) and Diabetes UK (Clinical Research Fellowship 10/0003985). Research clinics were held at the Wellcome Trust Clinical Research Facility and Princess Alexandra Eye Pavilion in Edinburgh. EHDPS was funded by the Medical Research Council and by the Chief Scientist Office of Scotland (Project Grant CZB/4/672). DNA standardisation was conducted at the Genetics Core of the Wellcome Trust Clinical Research Facility in Edinburgh. CaPS was funded by the Medical Research Council and undertaken by the former MRC Epidemiology Unit (South Wales). The DNA bank was established with funding from a MRC project grant. The data archive is maintained by the University of Bristol. The funders had no role in study design, data collection and analysis, decision to publish, or preparation of the manuscript."

