# Supplementary material for: Population Genomics of Cardiometabolic Traits: Design of the University College London-London School of Hygiene and Tropical Medicine-Edinburgh-Bristol (UCLEB) Consortium
Source: PLoS One. 2013 Aug 20;8(8):e71345. doi: 10.1371/journal.pone.0071345 (PMC3748096; doi:10.1371/journal.pone.0071345)
Supplement: Figure S1 — UCLEB workflow. (DOCX) [file pone.0071345.s001.docx]

**Figure S1. UCLEB workflow**


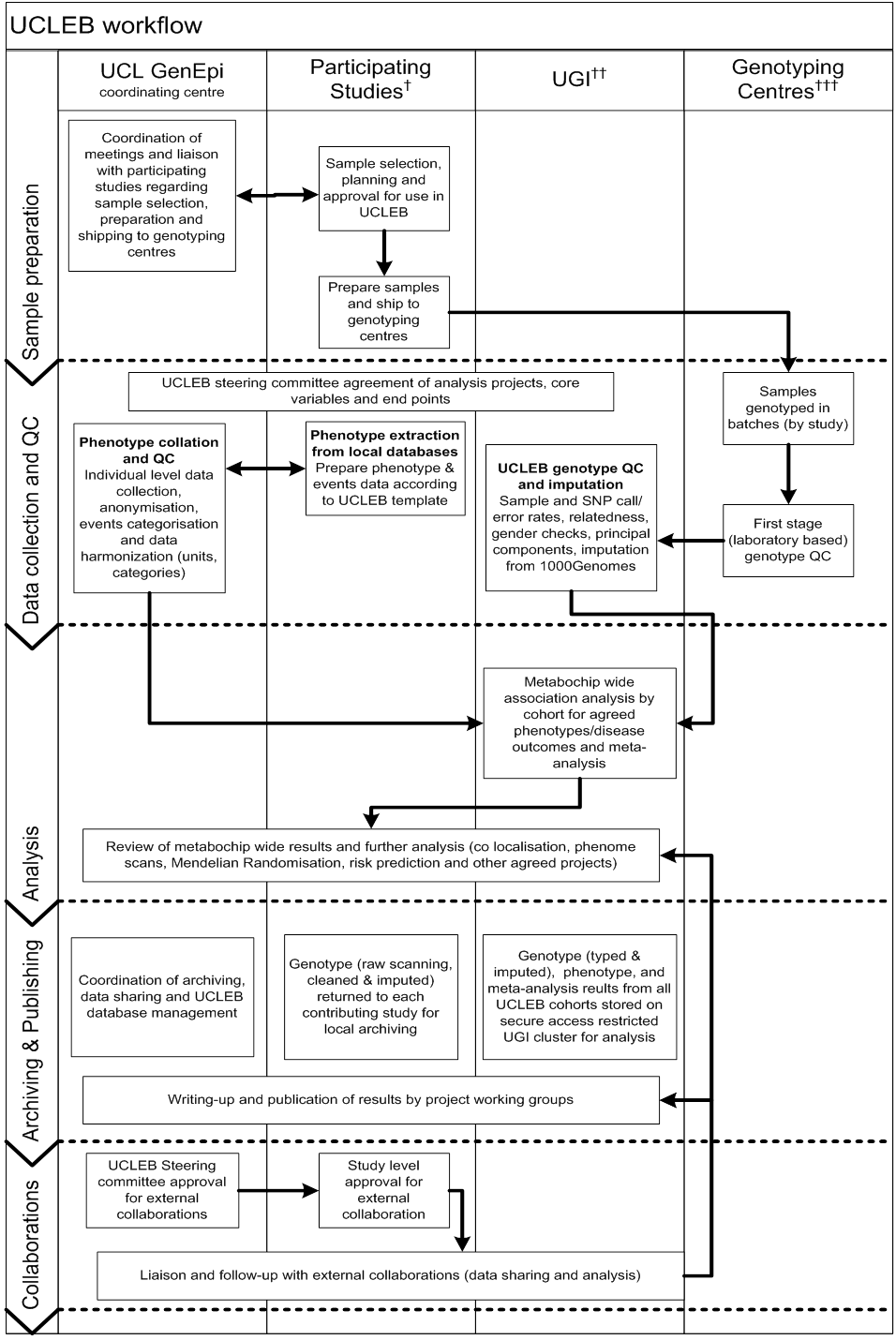


^†^Includes studies being genotyped under UCLEB: BRHS, BWHHS, CaPS, ET2DS, ELSA and MRC NSHD. Remaining studies either already have Metabochip data or will be used as replication cohorts.

^††^ UGI is the UCL Genetics Institute.

^†††^Three genotyping centres were used; UCL Genomics (CaPS, BWHHS and ELSA), Bart’s Genome centre (ET2DS, BRHS) and Cambridge Genomic Services (MRC NSHD). Studies with existing metabochip data were genotyped at Welcome Trust Edinburgh (EAS) and Cambridge Genomic Services (1958BC). WHII was typed at both Cambridge Genomic Services and UCL Genomics.
