# Supplementary figures and images for: Population Genomics of Cardiometabolic Traits: Design of the University College London-London School of Hygiene and Tropical Medicine-Edinburgh-Bristol (UCLEB) Consortium
Source: PLoS One. 2013 Aug 20;8(8):e71345. doi: 10.1371/journal.pone.0071345 (PMC3748096; doi:10.1371/journal.pone.0071345)

**Figure S2. Prioritised analyses in UCLEB**


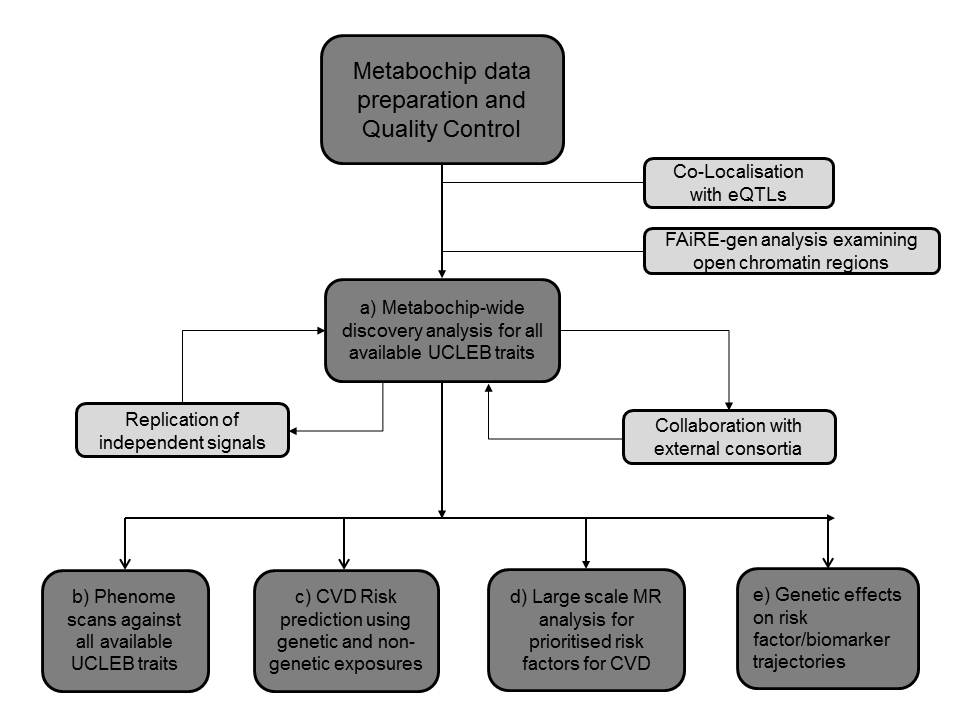

Supplement: Figure S2 — Prioritised analyses in UCLEB. (DOCX) [file pone.0071345.s002.docx]
