# Supplementary material for: Population Genomics of Cardiometabolic Traits: Design of the University College London-London School of Hygiene and Tropical Medicine-Edinburgh-Bristol (UCLEB) Consortium
Source: PLoS One. 2013 Aug 20;8(8):e71345. doi: 10.1371/journal.pone.0071345 (PMC3748096; doi:10.1371/journal.pone.0071345)
Supplement: Table S1 — a: Power for discovery for quantitative traits. Table S1b: Power for translation for quantitative traits (DOCX) [file pone.0071345.s003.docx]

**Table S1a. Power for discovery for quantitative traits**

|  | **% variance explained** | | | |
| --- | --- | --- | --- | --- |
| **N** | **0.5** | **1** | **2** | **5** |
| 2500 | 6.8 | 49.0 | 98.0 | 100 |
| 5000 | 49.0 | 98.0 | 100 | 100 |
| 10000 | 91.0 | 100 | 100 | 100 |
| 20000 | 100 | 100 | 100 | 100 |

**Table S1b. Power for translation for quantitative traits**

|  | **% variance explained** | | |
| --- | --- | --- | --- |
| **N** | **0.5** | **1** | **2** |
| 2500 | 36.1 | 86.6 | 99.9 |
| 5000 | 86.6 | 100 | 100 |
| 10000 | 100 | 100 | 100 |
