# Supplementary material for: Population Genomics of Cardiometabolic Traits: Design of the University College London-London School of Hygiene and Tropical Medicine-Edinburgh-Bristol (UCLEB) Consortium
Source: PLoS One. 2013 Aug 20;8(8):e71345. doi: 10.1371/journal.pone.0071345 (PMC3748096; doi:10.1371/journal.pone.0071345)
Supplement: Table S2 — a: Power for discovery for common alleles. Table S2b: Power for translation for common alleles. Table S2c: Power for discovery for rare alleles. Table S2d: Power for translation for rare alleles. (DOCX) [file pone.0071345.s004.docx]

**Table S2a. Power for discovery for common alleles**

|  | **MAF=10%** | | | **MAF=20%** | | | **MAF=30%** | | | **MAF=50%** | | |
| --- | --- | --- | --- | --- | --- | --- | --- | --- | --- | --- | --- | --- |
|  |  | **OR** |  |  | **OR** |  |  | **OR** |  |  | **OR** |  |
| **N** | **1.1** | **1.2** | **1.3** | **1.1** | **1.2** | **1.3** | **1.1** | **1.2** | **1.3** | **1.1** | **1.2** | **1.3** |
| 10000 | 0 | 2.7 | 28.9 | 0.2 | 15.8 | 78.5 | 0.4 | 31.8 | 94.2 | 0.6 | 44.8 | 98.2 |
| 20000 | 0.3 | 23.2 | 87.7 | 1.8 | 72.3 | 99.9 | 4.3 | 91.4 | 100 | 7.3 | 97.1 | 100 |
| 30000 | 1.0 | 57.1 | 99.3 | 7.1 | 96.5 | 100 | 16.3 | 99.7 | 100 | 25.6 | 100 | 100 |
| 40000 | 2.8 | 82.9 | 100 | 17.6 | 99.8 | 100 | 35.7 | 100 | 100 | 50.9 | 100 | 100 |
| 50000 | 6.0 | 94.8 | 100 | 32.3 | 100 | 100 | 57.0 | 100 | 100 | 73.2 | 100 | 100 |

**Table S2b. Power for translation for common alleles**

|  | **MAF=10%** | | | **MAF=20%** | | | **MAF=30%** | | | **MAF=50%** | | |
| --- | --- | --- | --- | --- | --- | --- | --- | --- | --- | --- | --- | --- |
|  |  | **OR** |  |  | **OR** |  |  | **OR** |  |  | **OR** |  |
| **N** | **1.1** | **1.2** | **1.3** | **1.1** | **1.2** | **1.3** | **1.1** | **1.2** | **1.3** | **1.1** | **1.2** | **1.3** |
| 10000 | 1.1 | 20.1 | 69.4 | 3.5 | 53.8 | 96.9 | 6.1 | 73.7 | 99.6 | 8.6 | 84.3 | 99.9 |
| 20000 | 4.7 | 63.7 | 98.7 | 16.1 | 95.4 | 100 | 27.7 | 99.3 | 100 | 37.4 | 99.9 | 100 |
| 30000 | 11.5 | 89.7 | 100 | 36.2 | 99.8 | 100 | 55.6 | 100 | 100 | 68.5 | 100 | 100 |
| 40000 | 21.1 | 97.9 | 100 | 57.3 | 100 | 100 | 77.5 | 100 | 100 | 87.6 | 100 | 100 |
| 50000 | 32.7 | 99.7 | 100 | 74.4 | 100 | 100 | 90.3 | 100 | 100 | 96.0 | 100 | 100 |

**Table S2c. Power for discovery for rare alleles**

|  | **MAF=0.1%** | | **MAF=1%** | | | **MAF=3%** | | | **MAF=5%** | | |  |
| --- | --- | --- | --- | --- | --- | --- | --- | --- | --- | --- | --- | --- |
|  | **OR** | | **OR** | | | **OR** | | | **OR** | | |  |
| **N** | **1.5** | **3** | | **1.5** | **3** | | **1.5** | **3** | | **1.5** | **3** | |
| 10000 | 0 | 2.6 | | 1.0 | 98.4 | | 20.3 | 100 | | 56.7 | 100 | |
| 20000 | 0 | 12.4 | | 7.4 | 100 | | 75.1 | 100 | | 98.2 | 100 | |
| 30000 | 0 | 29.1 | | 21.9 | 100 | | 96.7 | 100 | | 100 | 100 | |
| 40000 | 0 | 48.4 | | 41.7 | 100 | | 99.8 | 100 | | 100 | 100 | |
| 50000 | 0.2 | 65.9 | | 61.5 | 100 | | 100 | 100 | | 100 | 100 | |

**Table S2d. Power for translation for rare alleles**

|  | **MAF=0.1%** | | **MAF=1%** | | **MAF=3%** | | **MAF=5%** | |  |
| --- | --- | --- | --- | --- | --- | --- | --- | --- | --- |
|  | **OR** | | **OR** | | **OR** | | **OR** | |  |
| **N** | **1.5** | **3** | **1.5** | **3** | **1.5** | **3** | **1.5** | **3** | |
| 10000 | 0.3 | 14.1 | 9.3 | 99.9 | 57.2 | 100 | 88.2 | 100 | |
| 20000 | 0.7 | 38.8 | 32.8 | 100 | 95.5 | 100 | 99.9 | 100 | |
| 30000 | 1.2 | 62.5 | 59.0 | 100 | 99.8 | 100 | 100 | 100 | |
| 40000 | 1.8 | 79.7 | 78.7 | 100 | 100 | 100 | 100 | 100 | |
| 50000 | 2.7 | 89.9 | 90.3 | 100 | 100 | 100 | 100 | 100 | |
